# Intratumoural and peripheral blood lymphocyte subsets in patients with metastatic renal cell carcinoma undergoing interleukin-2 based immunotherapy: association to objective response and survival

**DOI:** 10.1038/sj.bjc.6600437

**Published:** 2002-07-02

**Authors:** F Donskov, K M Bennedsgaard, H von der Maase, N Marcussen, R Fisker, J J Jensen, P Naredi, M Hokland

**Affiliations:** Department of Oncology, Aarhus University Hospital, Denmark; Institute of Pathology, Aarhus University Hospital, Denmark; Department of Radiology, Aarhus University Hospital, Denmark; Department of Surgery, Umeaa University Hospital, Sweden; Institute of Medical Microbiology and Immunology, University of Aarhus, Denmark

**Keywords:** renal cell carcinoma, natural killer cell, lymphocytes, interleukin-2, prognostic factors

## Abstract

The aim of the present study was to analyse lymphocyte subsets in consecutive peripheral blood samples and consecutive tumour tissue core needle biopsies performed before and during interleukin-2 based immunotherapy, and to correlate the findings with objective response and survival. Twenty-six patients with metastatic renal cell carcinoma were treated with low dose s.c. interleukin-2, interferon-α and histamine. A total of 250 blood samples and 62 core needle biopsies from 23 and 19 of these patients, respectively, were analysed. After 2 weeks of treatment, a significant positive correlation between absolute number of peripheral blood lymphocytes (*P*=0.028), CD3 (*P*=0.017), CD57 (*P*=0.041) and objective response was demonstrated. There was no correlation between any peripheral blood leukocyte subsets and survival. Cytotoxicity of peripheral blood mononuclear cells was not correlated to objective response or survival. Within the tumour tissue at baseline, a significant positive correlation between CD4 (*P*=0.027), CD8 (*P*=0.028), CD57 (*P*=0.007) and objective response was demonstrated. After one month of immunotherapy, a significant positive correlation between intratumoral CD3 (*P*=0.026), CD8 (*P*=0.015), CD57 (*P*=0.009) and objective response was demonstrated. A significant positive correlation between intratumoral baseline CD4 (*P*=0.047), baseline CD57 (*P*=0.035), CD3 at one month (*P*=0.049) and survival was demonstrated. These data provide novel *in vivo* evidence of the possible contribution of lymphocyte subsets in the tumour reduction in responding patients during interleukin-2 based immunotherapy. Confirmation of the results requires further studies including a larger number of patients.

*British Journal of Cancer* (2002) **87**, 194–201. doi:10.1038/sj.bjc.6600437 www.bjcancer.com

© 2002 Cancer Research UK

## 

Manipulating the immune system by interleukin-2 (IL-2) based immunotherapy may induce durable tumour regression in metastatic renal cell carcinoma (mRCC) (Minasian *et al*, 1993; Fyfe *et al*, 1995; Jeal and Goa, 1997; Bordin *et al*, 2000; Fisher *et al*, 2000; Negrier *et al*, 2000). However, only a minority of patients benefit from treatment and consequently, many efforts have been made to assess parameters, which may predict objective response and survival in order to select those patients who most likely will benefit from treatment.

Our understanding of the mechanisms by which IL-2 and interferon-alpha (IFN-α) mediate their antineoplastic actions is incomplete. Based on peripheral blood sample analyses during the last 15 years, it is still unclear whether lymphocyte subsets during immunomodulative therapy in mRCC have an impact on objective response and survival (Bukowski, 1997; Jeal and Goa, 1997; Hernberg, 1999). Several studies have demonstrated increased number of lymphocytes (Palmer *et al*, 1993; Lissoni *et al*, 1994; Bordin *et al*, 2000), lymphocyte subsets (Wersall and Mellstedt, 1995; Gohring *et al*, 1996) including natural killer (NK-) cells (Atzpodien *et al*, 1993; von Rohr *et al*, 1993) and increased cytotoxicity (von Rohr *et al*, 1993; Wersall and Mellstedt, 1995) in responding patients during immunotherapy, but no consistent findings have been demonstrated (Bukowski, 1997; Jeal and Goa, 1997). No peripheral blood lymphocyte subset has been correlated to favourable survival. Moreover, knowledge about lymphocyte subsets within metastatic tumour tissue is scarce. In mRCC, only one study has focused on the immunological changes locally at the site of metastatic tumour during immunotherapy (Bukowski *et al*, 1997). In that study, however, biopsies were not performed systematically and no significant correlations to objective response or survival were found.

In an attempt to identify predictive factors for objective response and prognostic factors for survival, we have monitored lymphocyte subsets in as well consecutive peripheral blood samples as consecutive tumour tissue core needle biopsies in patients with mRCC undergoing immunotherapy with IL-2, IFN-α and histamine.

## MATERIALS AND METHODS

### Patients and samples

Twenty-six patients with metastatic renal cell carcinoma at the Department of Oncology, Aarhus University Hospital were enrolled in a multi-centre prospective phase II trial of s.c. IL-2, IFN-α and histamine dihydrochloride between February 1999 and February 2000 (Donskov *et al*, 2002). The treatment plan consisted of one priming-week of IFN-α and up to nine treatment cycles of interferon-α (human leukocyte IFN-α, Interferon Alfanative®, BioNative, Sweden) 3.0 million IU as a fixed dose, s.c. once daily, 7 days per week throughout the study; interleukin-2 (aldesleukin, rIL-2, Proleukin®, Chiron, The Netherlands) 2.4 million IU per m^2^, s.c., two times daily 5 days per week, weeks 1 and 2 every month; and histamine dihydrochloride (Ceplene™, Maxim Pharmaceuticals Inc, San Diego, USA) 1.0 mg in 1.0 ml by 20 min slow s.c. injections, two times daily, 5 days per week throughout the study. The addition of histamine was based on a new concept of how to increase the efficacy of IL-2 and IFN-α. However, by use of the present low dose regimen of IL-2 and IFN-α, histamine did not appear to add efficacy with respect to response (Donskov *et al*, 2002). Standard WHO criteria were used for classifying objective response (Donskov *et al*, 2002). All patients had clear cell carcinoma except one, who had a papillary renal cell carcinoma. This patient achieved stable disease (SD).

Of the 26 patients, 24 gave written informed consent for consecutive blood samples and core needle biopsies. One patient did not complete one course of therapy because of toxicity and was not evaluable for response. This patient was excluded from all further analysis. One patient was excluded from the core needle biopsy analysis as he had only fine needle biopsies performed. Thus, a total of 23 patients were evaluable for consecutive blood samples and 22 patients were evaluable for consecutive core needle tumour biopsies. Patient characteristics are given in Table 1

**Table 1:**
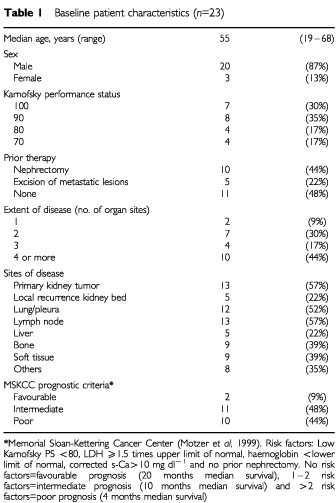
Baseline patient characteristics (*n*=23)

.

Blood samples were obtained at baseline, before start of week 1, 2 and 3 and thereafter before start of every second week throughout treatment. A total of 250 blood samples from 23 patients were evaluated (Table 2a

**Table 2a:**
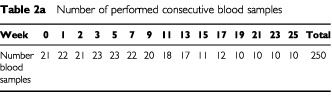
Number of performed consecutive blood samples

). Core needle biopsies (18G cutting needle) were collected by standard ultrasound-guided procedures (Jennings *et al*, 1989). Core needle biopsies were performed at baseline and before start of weeks 5, 12, 19, 24, 31 and 36 if possible. A total of 90 core needle biopsies in 22 patients were performed at different metastatic sites (subcutis, pleura, abdominal muscle, abdominal soft tissue, lymph node, liver and kidney). However, only 62 core needle biopsies in 19 patients were evaluable (Table 2b

**Table 2b:**
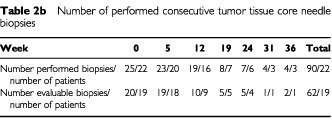
Number of performed consecutive tumor tissue core needle biopsies

). There were three non-evaluable patients, two because of insufficient tumour tissue in the biopsies and one because of necrosis in all biopsies at all time points. One patient had only a baseline biopsy performed (withdrawal of consent). Nineteen of the 90 biopsies (21%) were excluded because of necrosis. Moreover, in three of four responding patients, by week 12 and at following biopsy time points, tumour had disappeared (two patients) and biopsies contained only fibrotic materials (one patient). Thus, for the statistical analyses, only biopsies performed at baseline and at week 5 were used.

### Preparation of PBMCs

Peripheral blood mononuclear cells (PBMC) were isolated from lithium-heparinzed whole blood samples by Ficoll-Paque (Pharmacia Biotech, Uppsala, Sweden) gradient separation, washed twice and stored at −135°C until use.

### Flow cytometry

Surface phenotypes were determined by flow cytometry using a Coulter XL-2 flow cytometer (Coulter Electronics, UK). Data were analysed using Flow-Jo software (Treestar, San Carlos, CA, USA). Direct fluorochrome-conjugated antibodies (FITC or PE) were purchased from DAKO, Denmark (CD3, cat. no F0818; CD4, cat. no. F0766; CD8, cat. no. R0806 and CD20, cat. no. F0799) and Becton Dickinson, Denmark (CD56, cat. no. 347747 and CD57, cat. no. 347393). DAKO isotype controls were murine IgG1/FITC+IgG2a/RPE, cat. no. X 0949 and murine IgM/FITC, cat.no. X 0934. Leukogate was CD45/FITC+CD14 IgG2a/RPE, cat. no. FR 700, DAKO. A total of 2×10^5^ cells were incubated with antibody for 15 min at room temperature in the dark, washed twice with PBS containing 0.01% sodium azide and fixed in 0.25% formaldehyde. Flow cytometry was performed within 24 h.

### Cytotoxicity Assay

Cytolytic activity was determined by standard 4-h ^51^Cr-release assays. K562 tumour target cell pellets were suspended in 100 μCi of ^51^Cr (cat. no. NEZ030S, Dupharma, Kastrup, Denmark) and placed in a humidified incubator with 5% CO_2_ at 37°C for 1 h. Target cells were then washed free of excess ^51^Cr and plated in 96-well round-bottomed plate at 10^4^ cells well^−1^. Effectors (PBMC) were added to the wells at various effector-target ratios (40 : 1, 20 : 1 and 10 : 1). Wells containing target cells were used to determine spontaneous release. Wells containing target cells were mixed 10 times by the pipette and used to determine maximum target ^51^Cr release. Assay plates were incubated at 37°C with 5% CO_2_ for 4 h. A gamma counter (Cobra Autogamma Packard, Canberra Company, UK) was used to measure the radioactivity released from the lysed tumour cells. Per cent specific release was calculated as 100×(Experimental release−spontaneous release)/((Maximum release×0.8)−spontaneous release). The spontaneous release never exceeded 11% of the maximum release (in most experiments 4–8%). All blood samples from one patient were assayed within maximum 2 days.

### Immunohistochemistry

Sections (2 μm) of formalin-fixed, paraffin-embedded tissue were mounted on ChemMate slides (DAKO, Denmark) and dried 1 h at 60C. Following xylene deparaffinzation, rehydration through a series of graded alcohol and endogenous peroxidase blocking (0.5% hydrogen peroxidase in water for 30 min), antigen retrieval was performed by microwave oven heating (3×5 min at 850 W in Tris/EGTA retrieval buffer (pH 9.0)). Thereafter, slides were cooled to room temperature and placed in TBS. The following antibodies were used for 1 h of incubation: (a) CD3 (cat. no. A 0452, DAKO) 1 : 100; (b) CD4 (cat. no. NCL-CD4-1F6, NovoCastra) 1 : 50; (c) CD8 (cat. no. M 7103, DAKO) 1 : 100; (d) CD20 (cat. no. M 0755, DAKO) 1 : 500; (e) CD56 (cat. No. NCL-CD56-1B6, NovoCastra), 1 : 40; (f) CD57 (cat. no. 33251A, Pharmingen) 1 : 500; (g) CD79α (cat. no. M 7050, DAKO) 1 : 50. As second layer the Horseradish Peroxidase EnVision polymer (catalogue number K 4000/K 4002, DAKO, Denmark) was used for 30 min of incubation. Staining were visualised with diaminobenzidine tetrahydrochloride solution. Slides were counterstained in Mayer's haematoxylin and mounted with Aqutex (cat. no. 64912-50, KEBO-lab, Denmark). All dilutions were made in Antibody Diluent (cat. no. S 2022, DAKO). All staining were performed in a Techmate automate machine. Some of the staining was split to two staining sessions because of the amount of samples. A lymph node was used as a positive control. As negative controls, substitution of primary antibody with PBS and the following antibodies were used (h) IgG1 (cat. no. 33811A, Pharmingen) 1 : 50; (i) IgG2a (cat. no. 33031A, Pharmingen) 1 : 500; (j) IgG2b (cat. no. 33801A, Pharmingen) 1 : 500 and (k) IgM (cat. no. X 0942, DAKO) 1 : 20.

### Measuring intratumoural immune cells

A stereological examination was performed using a morphometric system consisting of a Olympus AH-3 microscope with a motorized stage, controlled by a computer for manual interactive counting on the computer screen (Gundersen *et al*, 1988). The software used was CAST-GRID v 1.09, developed by Olympus, Denmark. Each microscopic field of vision was projected onto the computer screen with a video camera, and the computer generated an unbiased counting frame in which the measurements were done. On the projected image of the section, the tumour area was encircled. Necrosis, artifacts and fibrous areas were omitted. The first field of vision was chosen at random, thereafter, the computer sampled systematically the following fields of vision within the entire encircled area. A number of 40 fields (4951 μm^2^ each) were counted. The entire core needle biopsy was assessed. Only a cell with staining restricted to the plasma membrane, a visible nucleus and located within the counting frame was counted as positive. The mean number of cells per mm^2^ tumour tissue was assessed for each patient. Staining was analysed blinded by one observer. For testing the reproducibility, CD3 and CD57 sections were ranked according to their number of immune cells and every eighth case was selected and counted blinded by a senior histopathologist (NM). A high level of reproducibility (Spearman's rho=0.9, *P*<0.0001) was found.

### Statistics

Results are expressed as median values. The relationship between baseline and on-treatment lymphocyte subsets and objective response was evaluated using the nonparametric Kruskal–Wallis test, followed by the Mann–Whitney rank-sum test for trend across ordered groups if appropriate. The relationship between baseline and on-treatment lymphocyte subsets and survival was evaluated using the method of Kaplan–Meier and the log-rank test. Dichotomy of the patients was done at the median value for each evaluated parameter. The median follow-up period was 32.3 months, (range 24.1–36.0 months). The survival data were updated 30th January 2002.

All reported *P*-values are two-sided. Statistical analyses were performed using SPSS v10.0.

## RESULTS

### Clinical treatment results

A total of 23 patients treated with low dose IL-2, IFN-α and histamine were evaluable for consecutive blood samples and tumour biopsies. Of these, four patients achieved partial remission (PR), eight patients achieved stable disease (SD) and 11 patients had progressive disease (PD). Median survival was 12.5 months (range 3.8−33.8+ months). Three patients (two with PR and one with SD) had no evidence of disease and were alive at 32.9+, 26.6+ and 33.8+ months, respectively, after subsequent resection of residual tumours. At the time of analysis, 17 patients had died, giving a censoring rate of 26.1%.

### Correlation between number of peripheral blood lymphocyte subsets and objective response

**Figure 1:**
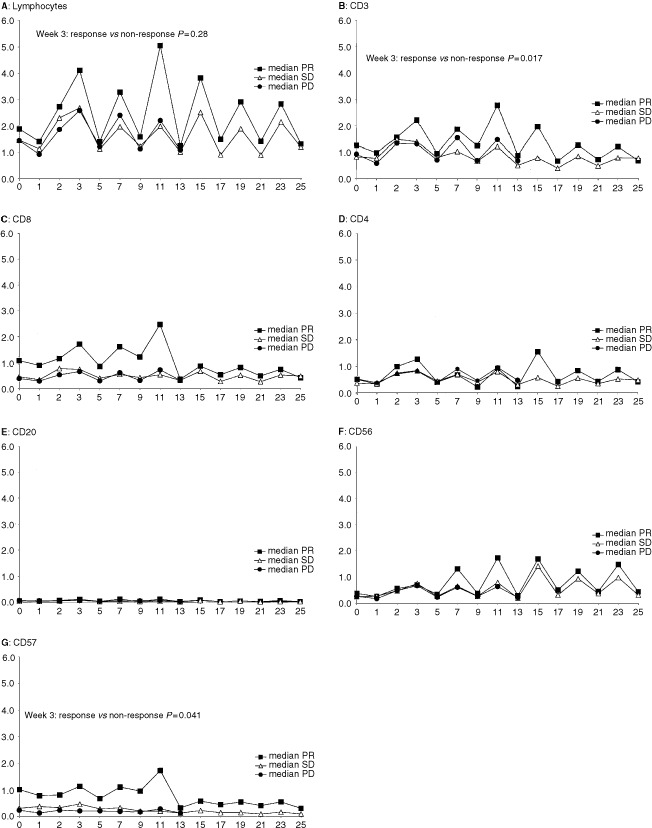
Monitoring of peripheral blood lymphocytes and lymphocyte subsets during interleukin-2 based immunotherapy. Median values for patients with partial responses (PR), stable disease (SD) and progressive disease (PD) are shown for (**A**): lymphocytes, (**B**): T-cells (CD3), (**C**): cytotoxic T-cells (CD8), (**D**): helper T-cells (CD4), (**E**): B-cells (CD20), (**F**): CD56 NK-cells and (**G**): CD57 NK-cells, respectively. Vertical and horizontal axes, 10^9^ cells l^−1^ and weeks during immunotherapy, respectively.

Baseline and on-treatment absolute number of lymphocytes and lymphocyte subsets defined by flow cytometry were examined and compared to responding (PR) and non-responding (SD+PD) patients. At baseline, no significant differences in responding patients compared to non-responding patients were demonstrated. Changes from baseline in the absolute number of lymphocytes and CD3 T-cells were detected in all patients. After 2 weeks of treatment, the absolute number of lymphocytes (*P*=0.028), CD3 T-cells (*P*=0.017) and CD57 NK cells (*P*=0.041) was significantly higher in responding patients compared with non-responding patients, Figure 1

. No differences in responding and non-responding patients were seen after 2 and 4 weeks of treatment in absolute number of CD4 helper T-cells (*P*=0.11), CD8 cytotoxic T-cells (*P*=0.061), CD20 B-cells (*P*=0.31) and CD56 NK-cells (*P*=0.61), Figure 1.

### Correlation between NK cell cytotoxicity and objective response

**Figure 2:**
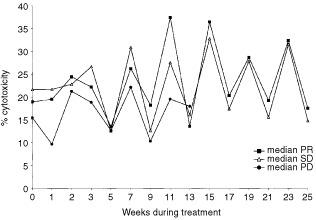
Cytotoxicity against K562 targets for patients with partial response (PR), stable disease (SD) and progressive disease (PD). Data points represent median cytotoxicity at an effector : target ratio of 40 : 1.

NK cell cytotoxicity defined by ^51^Chromium-release assay was monitored before and during therapy, Figure 2

. When cytotoxicity of responding and non-responding patients at baseline and during therapy was compared, no significant differences were noted. However, whereas cytotoxicity of PD patients remained at the same level of about 20% during treatment, cytotoxicity of non-PD (PR+SD) patients increased significantly at week 7 (*P*=0.03) and week 11 (*P*=0.033) compared with PD patients.

### Correlation between number and activity of peripheral blood lymphocyte subsets and survival

NK cell cytotoxicity, baseline and on-treatment absolute number of peripheral blood lymphocytes and lymphocyte subsets were examined and compared with survival. However, no significant correlations to survival were demonstrated (data not shown).

### Correlation between intratumoural lymphocyte subsets and objective response

**Figure 3:**
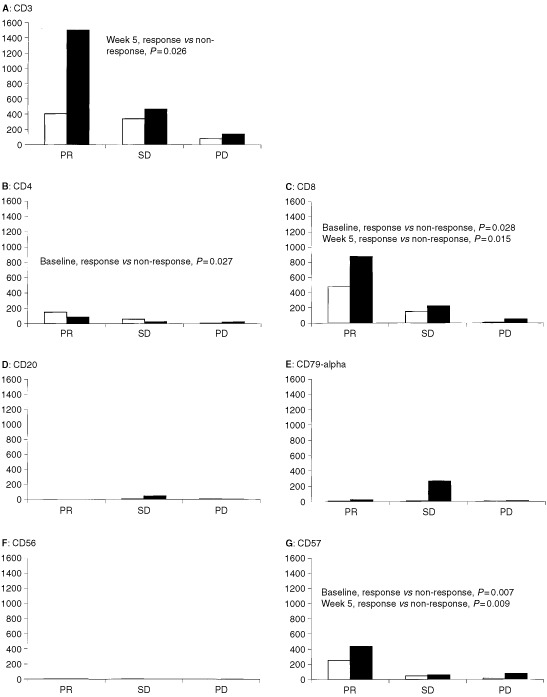
Intratumoural immune cells as predictors of response in patients with metastatic renal cell carcinoma at baseline (white bars) and after 1 month of treatment (black bars) with interleukin-2, interferon-alpha and histamine. (**A**): T-cells (CD3), (**B**): helper T-cells (CD4), (**C**): cytotoxic T-cells (CD8), (**D**): B-cells (CD20), (**E**) plasma cells (CD79-α), (**F**): CD56 NK-cells and (**G**): CD57 NK-cells. Horizontal and vertical axes, response to therapy and median number of cells per mm^2^ tumour tissue, respectively.

Baseline and on-treatment intratumoural lymphocyte subsets defined by immunohistochemistry were evaluated and correlated to objective response. At baseline, the number of CD4 cells per mm^2^ tumour tissue was significantly higher (*P*=0.027) in responding (PR) patients compared with non-responding (SD+PD) patients, Figure 3

. Moreover, the number of CD8 cells per mm^2^ tumour tissue and CD57 cells per mm^2^ tumour tissue at baseline was significantly higher (*P*=0.028 and *P*=0.007 respectively) in responding patients compared with non-responding patients. The number of intratumoral B-cells (CD20), plasma cells (CD79-α) and CD56 NK-cells were low, both at baseline and during treatment, and were not significantly correlated to objective response, Figure 3.

After 1 month of therapy, the number of CD3 cells per mm^2^ tumour tissue was significantly higher (*P*=0.026) in responding patients compared with non-responding patients. Moreover, the number of CD8 cells per mm^2^ tumour tissue and CD57 cells mm^2^ tumour tissue were significantly higher (*P*=0.015 and *P*=0.009, respectively) in responding patients compared with non-responding patients.

**Figure 4:**
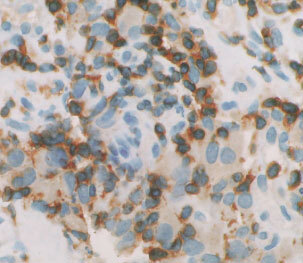
Intratumoural accumulation of CD3 T-cells in a responding patient after one month of immunotherapy. A clear cell-to-cell contact between CD3 T-cells (brown staining restricted to the plasma membrane) and tumour target cells (diffusely cytoplasmic brown staining) was noted. Magnification×40.

A clear cell-to-cell contact between immune effector cells and tumour target cells was noted in biopsies during immunotherapy, Figure 4

.

### Correlation between tumour-infiltrating lymphocyte-subsets and survival

**Figure 5:**
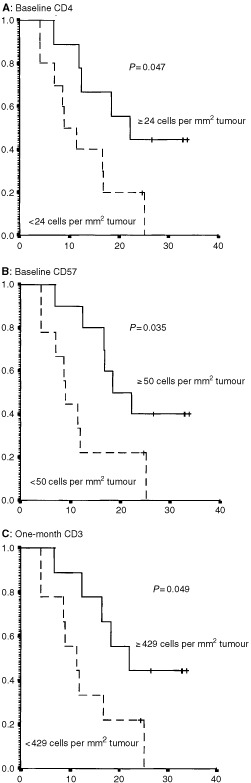
Intratumoural immune cells as prognostic factors for survival. Kaplan–Meier plots concerning survival according to (**A**): intratumoural baseline helper T-cells (CD4), (**B**): intratumoural baseline CD57+ natural killer cells and (**C**): intratumoural one-month T-cells (CD3) in patients with metastatic renal cell carcinoma treated with interleukin-2, interferon-α and histamine. Cutoff points were median values for each parameter. Vertical and horizontal axes, the survival probability and months of follow-up, respectively.

We also studied the influence of intratumoural lymphocyte subsets on survival. Intratumoural baseline CD4 (*P*=0.047), baseline CD57 (*P*=0.035) and one month CD3 (*P*=0.049) were all statistically significantly associated to survival, Figure 5

. Median survival of patients with ⩾24 CD4 cells per mm^2^ tumour tissue at baseline was 22.2 months compared to 8.9 months for patients with <24 CD4 cells per mm^2^ tumour tissue. For patients with more or less than 50 CD57 cells per mm^2^ tumour tissue at baseline, median survival was 18.4 months and 8.9 months, respectively. Furthermore, median survival of patients with ⩾429 CD3 cells per mm^2^ tumour tissue after one month of therapy was 22.2 months compared to 11.5 months for patients with <429 CD3 cells per mm^2^ tumour tissue. The numbers of intratumoral CD20, CD79-α and CD56 were not correlated to survival.

## DISCUSSION

This is to our knowledge the first study in patients with mRCC undergoing IL-2 based immunotherapy that demonstrates positive correlation between number of intratumoral lymphocyte subsets and both objective response and survival. Moreover, we demonstrate that the composition of peripheral blood lymphocyte subsets reflects the composition of intratumoral lymphocyte subsets. However, more detailed information is obtained from the intratumoral studies.

Little is known about how an effective antitumor response is orchestrated. Our results demonstrate that immune effector cells localise to sites of tumour in responding patients and that both CD4 and CD8 T-cell subsets are requisite for the response to IL-2 based immunotherapy. This is in accordance with findings in metastatic malignant melanoma treated with IL-2 (Rubin *et al*, 1989) and IFN-α (Hakansson *et al*, 1996; 2001). Thus, there seems to be a causative relationship between intratumoral lymphocyte subsets and both response and survival, as indicated by the fact that progressing patients had very few intratumoral lymphocyte subsets at both baseline and during treatment. On the other hand, our results should primarily be considered as hypotheses generation although all tested parameters were predefined. Consequently, our results have to be confirmed in subsequent studies.

NK cells are believed to contribute to the clinical efficacy of cancer immunotherapy using IL-2 in humans (Brittenden *et al*, 1996). However, NK cells show phenotypic heterogeneity in the peripheral blood (Jonges *et al*, 2001). Thus, the CD56 molecule is present on almost all NK cells, whereas the CD57 molecule is only present on a subpopulation of the total NK cells (Brittenden *et al*, 1996; Jonges *et al*, 2001). It is suggested that the CD57 antigen is only present in a mature subset of NK cells (Garcia-Sanz *et al*, 1996). Although there were no detectable intratumoral CD56 cells at baseline or during immunotherapy, as also reported in metastatic malignant melanoma (Rubin *et al*, 1989), we demonstrated that mononuclear cells expressing the CD57 molecule in both peripheral blood and tumour tissue were requisite for response. In addition, baseline intratumoural CD57 did influence on survival. Presence of intratumoral CD57 cells was also associated with favourable survival in studies of colorectal cancer (Coca *et al*, 1997) and gastric cancer (Ishigami *et al*, 2000).

Only few studies have monitored the *in vivo* cytotoxicity of NK cells during immunotherapy. To our knowledge, all studies have failed to detect any significant differences in K562-cytotoxicity between responding and non-responding patients, as our study also did. A few trials, however, have reported increased LAK cell activity in responding patients compared to non-responding patients (Krigel *et al*, 1990; von Rohr *et al*, 1993; Wersall and Mellstedt, 1995).

The humoural immune system, represented by B-cells and antibody-forming plasma cells were only present in peripheral blood and tumour tissue at low levels and were not correlated with response or survival. This finding was in accordance with most other studies (Bukowski, 1997; Bukowski *et al*, 1997) except a single study by Gohring *et al* (1996) demonstrating that baseline number of peripheral blood B-cells correlated with clinical response.

Our study suggests that tumour accumulation of specific immune cells (T cells) and non-specific immune cells (CD57 NK cells) were requisite for both objective response and survival. Thus, an effective antitumor response might be orchestrated with participation of cells with different functions and from different parts of the immune system.

All statistical differences in peripheral blood and tumour tissue lymphocyte subsets have the same direction, and it is thus unlikely that the significant findings are artefacts due to multiple comparisons.

In order to avoid sampling bias in measuring intratumoral immune cells, we used stereological examination (Gundersen *et al*, 1988), although most immunohistochemistry studies have used semiquantitive scoring techniques. Using this random and systematic sampling technique with an unbiased counting frame, a high level of reproducibility was found, as has also been demonstrated by others (Gundersen *et al*, 1988; Hansen *et al*, 1998). However, this technique is associated with considerable workload and time consumption. A special problem should be mentioned. Whereas patients with PD had very few and easily detectable intratumoural lymphocyte subsets, responding patients had large numbers of lymphocyte subsets, which in several cases made enumerations difficult. Moreover, whereas tumours in patients with PD remained unchanged, tumours in responding patients loosened and fibrotic areas developed. According to our counting rules, necrotic and fibrotic areas were avoided and only a cell with staining restricted to the plasma membrane, a visible nucleus and located within the counting frame was counted as positive. Thus, we may have underestimated the immune infiltrate in responding patients.

Differences in immunohistochemistry techniques, antibodies used, assay sensitivity and laboratory skills may influence the staining results. Consequently, only well-known and commercially available antibodies were used. Moreover all immunostainings were performed in an automatic staining machine and a two-layer EnVision technique was used, thereby allowing all slides stained in one or two turns. Thus, day-to-day variation was reduced and variation contributed by laboratory processing was minimized.

One of the main difficulties in interpreting studies on lymphocyte subsets and natural cytotoxicity in patients with cancer is the wide biologic variation existing in lymphocyte subsets number and activity in patients and healthy controls. Therefore, to make meaningful comparisons a larger number of subjects are required. In the present study, 24 out of 26 treated patients (92%) gave written informed consent for consecutive core needle biopsies and blood samples. Nineteen patients (79%) had evaluable biopsies. In comparison, Bukowski *et al* (1997) performed needle biopsies or open biopsies from 17 out of 36 treated patients (47%) during immunotherapy. Rubin *et al* (1989) performed biopsies on 82 patients but only 48 patients (59%) had evaluable biopsies. Thus, we succeeded to include a high frequency of patients into the biopsy part of the study. Despite that, it should be emphasized that our results are based on very few patients, including only four partial and no complete responders.

In conclusion, these data provide novel *in vivo* evidence of the possible contribution of lymphocyte subsets in the tumour reduction in responding patients during interleukin-2 based immunotherapy. Confirmation of the results requires further studies including a larger number of patients.

## References

[bib1] AtzpodienJKirchnerHKorferAHadamMSchomburgAMenzelTDeckertMFranzkeAVolkenandtMDallmannI1993 Expansion of peripheral blood natural killer cells correlates with clinical outcome in cancer patients receiving recombinant subcutaneous interleukin-2 and interferon-alpha-2 Tumour Biol 14 354359 8265981 10.1159/000217850

[bib2] BordinVGianiLMeregalliSBukovecRVaghiMMMandalaMPaolorossiFArdizzoiaATanciniGBarniSFrigerioFFumagalliLBordoniAValsuaniGDi FeliceGLissoniP2000 Five-year survival results of subcutaneous low-dose immunotherapy with interleukin-2 alone in metastatic renal cell cancer patients Urol Int 64 38 10782024 10.1159/000030473

[bib3] BrittendenJHeysSDRossJEreminO1996 Natural killer cells and cancer Cancer 77 12261243 8608497 10.1002/(sici)1097-0142(19960401)77:7<1226::aid-cncr2>3.0.co;2-g

[bib4] BukowskiRM1997 Natural history and therapy of metastatic renal cell carcinoma: the role of interleukin-2 Cancer 80 11981220 9317170 10.1002/(sici)1097-0142(19971001)80:7<1198::aid-cncr3>3.0.co;2-h

[bib5] BukowskiRMOlenckiTWangQPeereboomDBuddGTElsonPSandstromKTuasonLRaymanPTubbsRMcLainDKleinENovickAFinkeJ1997 Phase II trial of interleukin-2 and interferon-alpha in patients with renal cell carcinoma: clinical results and immunologic correlates of response J Immunother 20 301311 9220320 10.1097/00002371-199707000-00007

[bib6] CocaSPerez-PiquerasJMartinezDColmenarejoASaezMAVallejoCMartosJAMorenoM1997 The prognostic significance of intratumoral natural killer cells in patients with colorectal carcinoma Cancer 79 23202328 9191519 10.1002/(sici)1097-0142(19970615)79:12<2320::aid-cncr5>3.0.co;2-p

[bib7] DonskovFvon der MaaseHHenrikssonRStiernerUWersallPNellemannHHellstrandKEngmanKNarediP2002 Outpatient treatment with subcutaneous histamine dihydrochloride in combination with interleukin-2 and interferon-alpha in patients with metastatic renal cell carcinoma: results of an open single-armed multicentre phase II study Ann Oncol 13 441449 11996477 10.1093/annonc/mdf049

[bib8] FisherRIRosenbergSAFyfeG2000 Long-term survival update for high-dose recombinant interleukin-2 in patients with renal cell carcinoma Cancer J Sci Am 6Suppl 1S55S57 10685660

[bib9] FyfeGFisherRIRosenbergSASznolMParkinsonDRLouieAC1995 Results of treatment of 255 patients with metastatic renal cell carcinoma who received high-dose recombinant interleukin-2 therapy J Clin Oncol 13 688696 7884429 10.1200/JCO.1995.13.3.688

[bib10] Garcia-SanzRGonzalezMOrfaoAMoroMJHernandezJMBorregoDCarneroMCasanovaFBarezAJimenezRPorteroJASan MiguelJF1996 Analysis of natural killer-associated antigens in peripheral blood and bone marrow of multiple myeloma patients and prognostic implications Br J Haematol 93 8188 8611480 10.1046/j.1365-2141.1996.4651006.x

[bib11] GohringBRiemannDRebmannUHeynemannHSchabelJLangnerJ1996 Prognostic value of the immunomonitoring of patients with renal cell carcinoma under therapy with IL-2/IFN-alpha-2 in combination with 5-FU Urol Res 24 297303 8931295 10.1007/BF00304780

[bib12] GundersenHJBendtsenTFKorboLMarcussenNMollerANielsenKNyengaardJRPakkenbergBSorensenFBVesterbyA1988 Some new, simple and efficient stereological methods and their use in pathological research and diagnosis APMIS 96 379394 3288247 10.1111/j.1699-0463.1988.tb05320.x

[bib13] HakanssonAGustafssonBKrysanderLHakanssonL1996 Tumour-infiltrating lymphocytes in metastatic malignant melanoma and response to interferon alpha treatment Br J Cancer 74 670676 8845294 10.1038/bjc.1996.420 PMC2074699

[bib14] HakanssonAGustafssonBKrysanderLHjelmqvistBRettrupBHakanssonL2001 Biochemotherapy of metastatic malignant melanoma. Predictive value of tumour-infiltrating lymphocytes Br J Cancer 85 18711877 11747328 10.1054/bjoc.2001.2169 PMC2364006

[bib15] HansenSGrabauDARoseCBakMSorensenFB1998 Angiogenesis in breast cancer: a comparative study of the observer variability of methods for determining microvessel density Lab Invest 78 15631573 9881956

[bib16] HernbergM1999 Lymphocyte subsets as prognostic markers for cancer patients receiving immunomodulative therapy Med Oncol 16 145153 10523794 10.1007/BF02906126

[bib17] IshigamiSNatsugoeSTokudaKNakajoACheXIwashigeHAridomeKHokitaSAikouT2000 Prognostic value of intratumoral natural killer cells in gastric carcinoma Cancer 88 577583 10649250

[bib18] JealWGoaKL1997 Aldesleukin (Recombinant Interleukin-2). A review of its pharmacological properties, clinical efficacy and tolerability in patients with renal cell carcinoma BioDrugs 7 285317 18020488 10.2165/00063030-199707040-00005

[bib19] JenningsPEDonaldJJCoralARodeJLeesWR1989 Ultrasound-guided core biopsy Lancet 1 13691371 2567382 10.1016/s0140-6736(89)92813-4

[bib20] JongesLEAlbertssonPvan VlierbergheRLEnsinkNGJohanssonBRvan de VeldeCJFleurenGJNannmarkUKuppenPJ2001 The phenotypic heterogeneity of human natural killer cells: presence of at least 48 different subsets in the peripheral blood Scand J Immunol 53 103110 11169213 10.1046/j.1365-3083.2001.00838.x

[bib21] KrigelRLPadavic-ShallerKARudolphARKonradMBradleyECComisRL1990 Renal cell carcinoma: treatment with recombinant interleukin-2 plus beta-interferon J Clin Oncol 8 460467 2407809 10.1200/JCO.1990.8.3.460

[bib22] LissoniPBarniSArdizzoiaACrispinoSPaolorossiFAndresMScardinoETanciniG1994 Prognostic factors of the clinical response to subcutaneous immunotherapy with interleukin-2 alone in patients with metastatic renal cell carcinoma Oncology 51 5962 8265104 10.1159/000227311

[bib23] MinasianLMMotzerRJGluckLMazumdarMVlamisVKrownSE1993 Interferon alfa-2a in advanced renal cell carcinoma: treatment results and survival in 159 patients with long-term follow-up J Clin Oncol 11 13681375 8315435 10.1200/JCO.1993.11.7.1368

[bib24] MotzerRJMazumdarMBacikJBergWAmsterdamAFerraraJ1999 Survival and prognostic stratification of 670 patients with advanced renal cell carcinoma J Clin Oncol 17 25302540 10561319 10.1200/JCO.1999.17.8.2530

[bib25] NegrierSMaralJDrevonMVinkeJEscudierBPhilipT2000 Long-term follow-up of patients with metastatic renal cell carcinoma treated with intravenous recombinant interleukin-2 in Europe Cancer J Sci Am 6Suppl 1S93S98 10685667

[bib26] PalmerPAAtzpodienJPhilipTNegrierSKirchnerHvon der MaaseHGeertsenPEversPLoriauxEOskamR1993 A comparison of 2 modes of administration of recombinant interleukin-2: continuous intravenous infusion alone versus subcutaneous administration plus interferon alpha in patients with advanced renal cell carcinoma Cancer Biother 8 123136 7804353 10.1089/cbr.1993.8.123

[bib27] RubinJTElwoodLJRosenbergSALotzeMT1989 Immunohistochemical correlates of response to recombinant interleukin-2- based immunotherapy in humans Cancer Res 49 70867092 2582450

[bib28] von RohrAGhoshAKThatcherNSternPL1993 Immunomodulation during prolonged treatment with combined interleukin-2 and interferon-alpha in patients with advanced malignancy Br J Cancer 67 163171 7678979 10.1038/bjc.1993.29 PMC1968230

[bib29] WersallPMellstedtH1995 Increased LAK and T cell activation in responding renal cell carcinoma patients after low dose cyclophosphamide, IL-2 and alpha-IFN Med Oncol 12 6977 8535664 10.1007/BF01676706

